# Pathological Significance and Prognostic Value of Surfactant Protein D in Cancer

**DOI:** 10.3389/fimmu.2018.01748

**Published:** 2018-08-06

**Authors:** Alessandro Mangogna, Beatrice Belmonte, Chiara Agostinis, Giuseppe Ricci, Alessandro Gulino, Ines Ferrara, Fabrizio Zanconati, Claudio Tripodo, Federico Romano, Uday Kishore, Roberta Bulla

**Affiliations:** ^1^Department of Life Sciences, University of Trieste, Trieste, Italy; ^2^Tumor Immunology Unit, Department of Health Sciences, Human Pathology Section, University of Palermo, Palermo, Sicily, Italy; ^3^Institute for Maternal and Child Health, IRCCS (Istituto di Ricovero e Cura a Carattere Scientifico) Burlo Garofolo, Trieste, Italy; ^4^Department of Medical, Surgical and Health Science, University of Trieste, Trieste, Italy; ^5^Biosciences, College of Health and Life Sciences, Brunel University London, Uxbridge, United Kingdom

**Keywords:** innate immunity, surfactant protein D, immune surveillance, bioinformatics analysis, immunohistochemistry, cancers, tumor microenvironment

## Abstract

Surfactant protein D (SP-D) is a pattern recognition molecule belonging to the Collectin (collagen-containing C-type lectin) family that has pulmonary as well as extra-pulmonary existence. In the lungs, it is a well-established opsonin that can agglutinate a range of microbes, and enhance their clearance *via* phagocytosis and super-oxidative burst. It can interfere with allergen–IgE interaction and suppress basophil and mast cell activation. However, it is now becoming evident that SP-D is likely to be an innate immune surveillance molecule against tumor development. SP-D has been shown to induce apoptosis in sensitized eosinophils derived from allergic patients and a leukemic cell line *via* p53 pathway. Recently, SP-D has been shown to suppress lung cancer progression *via* interference with the epidermal growth factor signaling. In addition, a truncated form of recombinant human SP-D has been reported to induce apoptosis in pancreatic adenocarcinoma *via* Fas-mediated pathway in a p53-independent manner. To further establish a correlation between SP-D presence/levels and normal and cancer tissues, we performed a bioinformatics analysis, using Oncomine dataset and the survival analysis platforms Kaplan–Meier plotter, to assess if SP-D can serve as a potential prognostic marker for human lung cancer, in addition to human gastric, breast, and ovarian cancers. We also analyzed immunohistochemically the presence of SP-D in normal and tumor human tissues. We conclude that (1) in the lung, gastric, and breast cancers, there is a lower expression of SP-D than normal tissues; (2) in ovarian cancer, there is a higher expression of SP-D than normal tissue; and (3) in lung cancer, the presence of SP-D could be associated with a favorable prognosis. On the contrary, at non-pulmonary sites such as gastric, breast, and ovarian cancers, the presence of SP-D could be associated with unfavorable prognosis. Correlation between the levels of SP-D and overall survival requires further investigation. Our analysis involves a large number of dataset; therefore, any trend observed is reliable. Despite apparent complexity within the results, it is evident that cancer tissues that produce less levels of SP-D compared to their normal tissue counterparts are probably less susceptible to SP-D-mediated immune surveillance mechanisms *via* infiltrating immune cells.

## Introduction

Surfactant protein D (SP-D) is a collagenous glycoprotein encoded by *SFTPD* gene belonging to the collectins family (1). Like other members of the collectin family, SP-D has a primary subunit structure that comprises of an N-terminal cysteine-rich region, a triple-helical collagen-like domain, an α-helical coiled neck domain, and a C-terminal C-type lectin domain [also called carbohydrate recognition domain (CRD)] (2). Each subunit of human SP-D comprises three identical polypeptide chains of 43 kDa, which is assembled into a tetrameric structure with four of the homotrimeric subunits linked *via* their N-terminal regions, but trimers, dimers, and monomers also exist. Tetrameric structures can undergo further oligomerization to give SP-D multimers that could contain up to 96 individual chains. SP-D was originally described in association with pulmonary surfactant; in the lung, it is synthesized and secreted by type II alveolar cells and non-ciliated bronchiolar epithelial cells. It has a key role in the maintenance of surfactant homeostasis by reducing surface tension (3). Reduced SP-D expression or genetic variations (single-nucleotide polymorphism) have been associated with an increased risk of respiratory diseases (4, 5).

Extra-pulmonary existence of SP-D has also been reported. SP-D is also expressed by epithelial cells lining various exocrine ducts, the mucosa of the gastrointestinal and genitourinary tracts, the nasal cavity, and in the brain (2). Furthermore, its presence has been demonstrated in healthy lacrimal gland, conjunctiva, cornea, and nasolacrimal duct samples (6). Other studies have shown the presence of SP-D in synovial fluid derived from patients with rheumatoid arthritis (7).

In addition to its role in surfactant homeostasis, SP-D has a critical function as a regulator of inflammation (3). It is involved in the recognition and neutralization of pathogens which promotes aggregation/agglutination and inhibition of microbial growth (8), SP-D has also been implicated in the clearance of necrotic and apoptotic cells (9). Thus, its function in the recognition of non-self and altered self makes it a potent and versatile humoral pattern recognition receptor (10–12). SP-D has also been described as a potent link between innate and adaptive immune mechanisms (13–15). Studies involving *in vivo* and *ex vivo* models of allergic inflammation revealed that SP-D can alleviate pulmonary hypersensitivity *via* suppression of IgE levels, promotion of Th2 to Th1 polarization (16), apoptosis induction in sensitized eosinophils *via* p53-mediated pathway (17), and inhibition of IgE synthesis by B cells (18). These studies highlighted a potential role of SP-D as an immune surveillance molecule. It has recently been shown that SP-D also plays a role in the control of lung cancer progression *via* epidermal growth factor (EGF) signaling (19). Very recently, Kaur et al. have shown that a recombinant fragment of human SP-D, composed of homotrimeric neck and C-type lectin domains, can induce apoptosis in pancreatic adenocarcinoma cell lines, such as Panc-1 (p53^mt^), MiaPaCa-2 (p53^mt^), and Capan-2 (p53^wt^), *via* Fas-mediated pathway (20).

In the current study, we performed a bioinformatics analysis in order to investigate whether SP-D can serve as a potential prognostic marker for human lung cancer. We extended our investigation to several non-pulmonary sites such as human gastric, breast, and ovarian cancer. We used the Oncomine dataset and the survival analysis platforms Kaplan–Meier plotter. Our results appear to suggest a likely pro-tumorigenic role of SP-D in gastric, breast, and ovarian cancers and an anti-tumor effect in lung cancer. Furthermore, we analyzed the presence of SP-D in normal and tumor human tissues *via* immunohistochemistry (IHC). Differential expression of SP-D was also investigated in human cells isolated from normal and tumor ovary tissues by real-time PCR. This *in silico* study, if validated *via* a retrospective study at the protein level, could be a step forward in ascertaining the importance of SP-D as a prognostic biomarker for different cancers.

## Materials and Methods

### Oncomine Database Analysis

The expression level of *SFTPD* gene in various types of cancer was analyzed using Oncomine,^1^ a cancer microarray database and web-based data mining platform from genome-wide expression analyses (21, 22). We compared the differences in mRNA level between normal tissue and cancer. The mRNA expression level in neoplastic tissues compared to the healthy tissues was obtained as the parameters of *p*-value < 0.001, fold change > all, and gene ranking in the top 10%. Information about the datasets used in this study is summarized in Table 1.

**Table 1:** Data characteristics used in the bioinformatics analysis.

Datasets	Study description	Experiment type
Bhattacharjee lung	139 lung adenocarcinoma, 21 squamous cell lung carcinoma, 20 lung carcinoid tumor, 6 small cell lung carcinoma, and 17 normal lung samples were analyzed on Affymetrix U95A microarrays. Sample data includes type, age, M stage, max tumor percentage, N stage, primary/metastatic, recurrence, sex, site of metastasis, smoking rate (packs per year), stage, survival, and T stage	mRNA
Hou lung	91 non-small cell lung carcinoma and 65 adjacent normal lung samples were analyzed. Sample data includes age, sex, cancer sample site, and survival	mRNA
Garber lung	67 lung carcinoma samples of various types and 6 normal lung samples were analyzed on cDNA microarrays. Sample data includes type, grade, TNM stage, and survival	mRNA
Cho gastric	65 gastric adenocarcinoma, 19 paired surrounding normal tissue, and 6 gastrointestinal stromal tumor samples were analyzed. Sample data includes age, grade, stage, TNM stage, sex, and subgroup	mRNA
DErrico gastric	31 paired gastric carcinoma and adjacent normal gastric mucosa and 7 unmatched gastric carcinoma samples were analyzed. Sample data includes microsatellite status, age, sex, and TNM stage	mRNA
Zhao breast	Normal breast (*n* = 3) and breast carcinoma (*n* = 61) samples were analyzed on cDNA microarrays. Sample data includes tumor percentage, age, E-Cadherin status, estrogen receptor status, grade, HER2 status, lymph node metastasis status, and progesterone receptor status	mRNA
TCGA breast	532 invasive breast carcinoma, 61 paired normal breast tissue, and 3 paired metastatic samples were analyzed. Sample data includes age, histology, TNM stage, ER/PR/ERBB2 status, sex, stage, and others. This dataset consists of Level 2 (processed) data from the TCGA data portal	mRNA
Curtis breast	1,992 breast carcinoma samples and 144 paired normal breast samples were analyzed for the METABRIC project. Sample data includes ER/PR/ERBB2 status, overall survival status and follow-up time, stage, grade, and others	mRNA
Yoshihara ovarian	43 ovarian serous adenocarcinomas and 10 normal peritoneum samples were analyzed. Sample data includes cancer sample site, stage, and sex	mRNA
TCGA ovarian	586 ovarian serous cystadenocarcinoma samples and 8 normal ovary samples were analyzed. Sample data includes age, stage, grade, survival, and others. This dataset consists of Level 2 (processed) data from the TCGA data portal	mRNA

*Abbreviations: TNM, Tumor-Nodes-Metastasis; HER2, Human Epidermal Growth Factor Receptor 2; ER, Estrogen Receptor; PR, Progesterone Receptor; ERBB, Erb-b2 receptor tyrosine kinase 2*.

### Kaplan–Meier Plotter Database Analysis

A Kaplan–Meier plotter database can assess the effect of 54,675 genes on survival using 10,461 cancer samples (5,143 breast, 1,816 ovarian, 2,437 lung, and 1,065 gastric cancer patients with a mean follow-up of 69/40/49/33 months) using probe sets on the HGU133 Plus 2.0 array. The prognostic significance of SP-D expression and survival in breast, ovarian, lung, and gastric cancer was analyzed by Kaplan–Meier plotter^2^ (23). The hazard ratio with 95% confidence intervals and logrank *p*-value was also computed.

### Patients and Specimens

Eight fresh clinical specimens (four normal ovarian epithelial tissues and four malignant ovarian epithelial tumor tissues) were obtained from the Department of Gynaecology of IRCCS “Burlo Garofolo”, in Trieste, Italy between 2016 and 2017. Cancer patients underwent laparoscopy for diagnosis of pelvic mass whereas control patients underwent laparoscopy for other indications. Tissue samples from patients were collected after informed consent following ethical approval by the Institutional Board of IRCCS “Burlo Garofolo”, Trieste, Italy.

### Immunohistochemical Analysis

For the immunohistochemical analysis, human normal and neoplastic tissues, including lung, breast, ovary, and stomach samples, were selected from the archives of the Department of Pathology, University of Palermo. Immunohistochemistry (IHC) was performed using a polymer detection method. Briefly, tissue samples were fixed in 10% v/v buffered formalin and then paraffin embedded. 4 µm-thick tissue sections were deparaffinized and rehydrated. The antigen unmasking technique was carried out using Novocastra Epitope Retrieval Solutions, pH 9 (Leica Biosystems) in a PT Link pre-treatment module (Dako) at 98°C for 30 min. Sections were then brought to room temperature and washed in PBS. After neutralization of the endogenous peroxidase with 3% v/v H_2_O_2_ and Fc blocking by a specific protein block (Novocastra, Leica Biosystems), samples were incubated overnight at 4°C with rabbit anti-human SP-D (dilution 1:300) polyclonal antibodies (MRC Immunochemistry Unit, Oxford, UK). Staining was revealed *via* polymer detection kit (Novocastra, Leica Biosystems) and AEC (3-amino-9-ethylcarbazole, Dako, Denmark) substrate-chromogen. Slides were counterstained with Harris Hematoxylin (Novocastra, Leica Biosystems). Sections were analyzed under the Axio Scope A1 optical microscope (Zeiss) and microphotographs were collected through the Axiocam 503 color digital camera (Zeiss) using the Zen2 software.

### Cell Isolation and Culture

Ovarian carcinoma cells (OvCa) and normal epithelial ovarian cells (OvEp) were isolated from biopsies derived from ovarian tissue. The tissue was finely minced with a cutter, incubated with a digestion solution composed by 0.5% trypsin (Sigma-Aldrich, Milan, Italy) and 50 µg/ml DNase I (Roche, Milan, Italy) in Hanks’ Balanced Salt solution containing 0.5 mM Ca^2+^Mg^2+^ (Sigma-Aldrich) overnight at 4°C. Next, the enzymatic solution was changed to collagenase type 1 (1.5 mg/ml) (Worthington Biochemical Corporation, DBA) diluted in Medium 199 with Hank’s salts (Euroclone Spa, Milan, Italy) for 30 min at 37°C. The digestion was blocked with 10% v/v fetal bovine serum (FBS; GIBCO, Life Technology) and the cell suspension was passed through a 100 µm pore filter (BD Biosciences, Italy). The cells were seeded in a 25 cm^2^ flask, coated with bovine gelatine, and cultured using Human Endothelial cells serum-free medium (HESF; Life Technologies), 10% heat-inactivated FBS supplemented with EGF (10 ng/ml), basic FGF (20 ng/ml) and Penicillin–Streptomycin (Sigma-Aldrich). Fresh medium was replaced every 2–3 days. The cells were maintained at 37°C in humidified atmosphere with 5% v/v CO_2_ and used at their fifth to eighth passage for *in vitro* experiments.

### RNA Isolation, cDNA Synthesis, and Quantitative Real-Time PCR (qPCR)

Total RNA was extracted from cells using EuroGOLD trifast (Euroclone), according to the manufacturer’s instructions, and reverse-transcribed as previously described (24). qPCR was carried out using a Rotor-Gene 6000 (Corbett, Qiangen, Ancona, Italy) using iQ SYBR Green Supermix (Applied Biosystems, Milan, Italy). The sequences of the primers used for amplification of TataBox Binding Protein (TBP) housekeeping gene are Forward 5′-GAGCCAAGAGTGAAGAACAGTC-3′; Reverse 5′-GCTCCCCACCATATTCTGAATCT-3′. The sequences of SP-D primers are Forward 5′-AGGCTGCTTTCCTGAGCATGAC-3′; Reverse 5′-CCATTGGTGAAGATCTCCACACAG-3′. The melting curve was recorded between 55 and 99°C with a hold every 2 s. The relative amount of gene production in each sample was determined by the Comparative Quantification method supplied as part of the Rotor Gene 1.7 software (Corbett Research) (25). The relative amount of each gene was normalized with TBP and expressed as arbitrary units (AU) considering 1 AU obtained from fully differentiated macrophage used as calibrator.

### Statistical Analysis

Survival curves were generated by the Kaplan–Meier plots. All results are displayed with *p* values from a logrank test. *p*-values < 0.05 were considered significant. Similarly, with Oncomine, the statistical significance of data (*p*-values) was provided by the program.

## Results

### Clinical Significance of SP-D Expression in Lung Cancer

**Figure 1:**
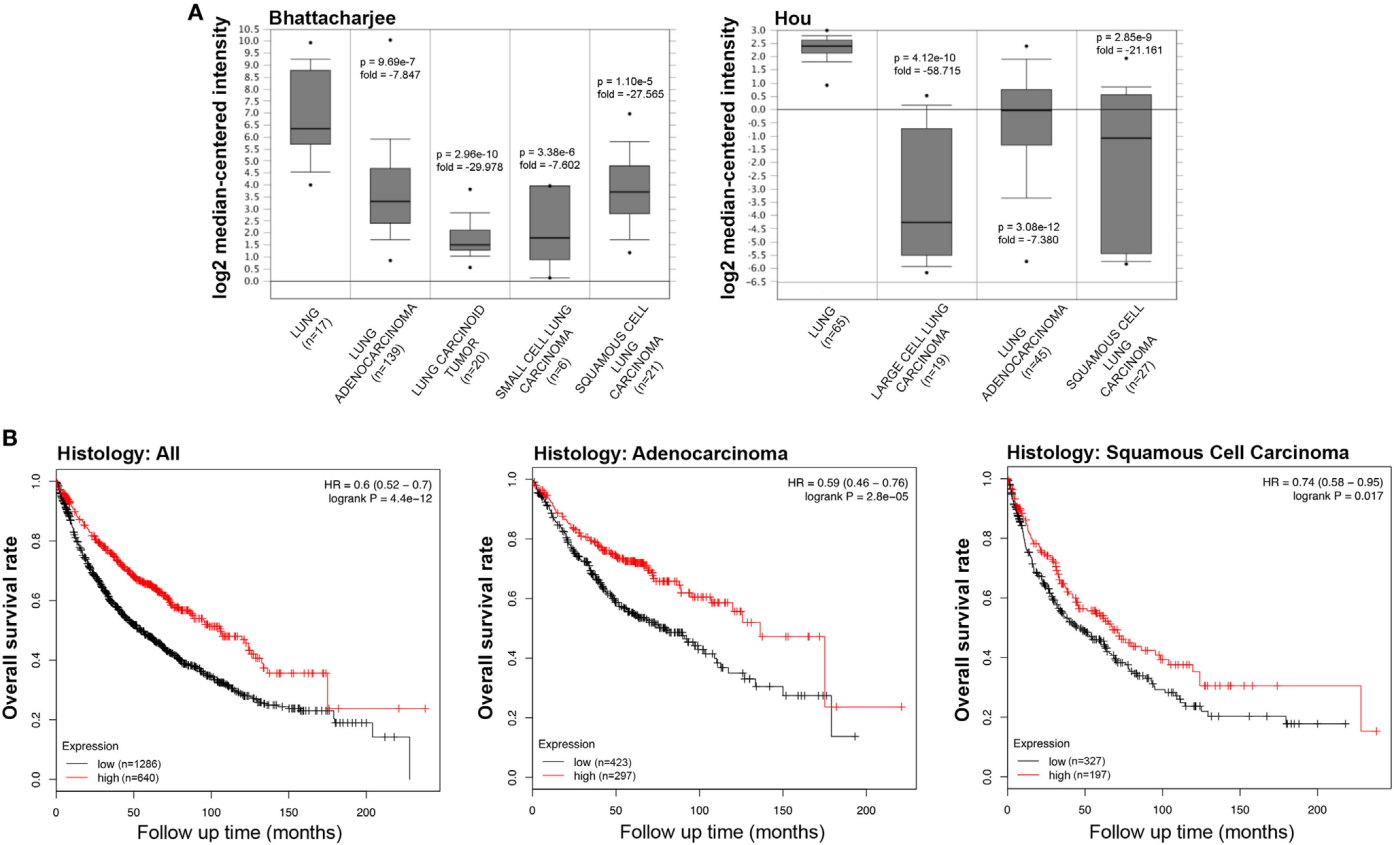
Pathological significance of SP-D expression in lung cancer. Bhattacharjee’s and Hou’s datasets were used for bioinformatics analysis to explore SP-D mRNA expression in the lung cancer. A lower SP-D mRNA expression was detectable in lung adenocarcinoma, squamous cell carcinoma, large cell carcinoma, small cell carcinoma, and tumor carcinoid than in normal lung tissue [**(A)** *p* < 0.05]. According to the data from Kaplan–Meir plotter, SP-D mRNA expression was positively related to an overall survival rate of the patients with lung cancer, even stratified into lung adenocarcinoma and squamous cell carcinoma [**(B)** *p* < 0.05]. Abbreviations: HR, hazard ratio; SP-D, surfactant protein D.

We initially compared the differences in the mRNA level of SP-D between neoplastic and healthy tissues using the Oncomine platform. While analyzing Bhattacharjee’s, Hou’s, and Garber’s datasets, we detected a significantly lower SP-D mRNA expression in lung adenocarcinoma, squamous cell carcinoma, large cell carcinoma, small cell carcinoma, and tumor carcinoid, compared to the normal lung tissue (Figure 1A, *p* < 0.05; Figure S1 in Supplementary Material, *p* < 0.05). We subsequently performed a bioinformatic analysis of SP-D mRNA expression using the Kaplan–Meier plotter dataset. As shown in Figure 1B, SP-D mRNA expression was positively related to an overall survival rate of the patients with lung cancer, stratified into lung adenocarcinoma and squamous cell carcinoma (*p* < 0.05).

**Figure 2:**
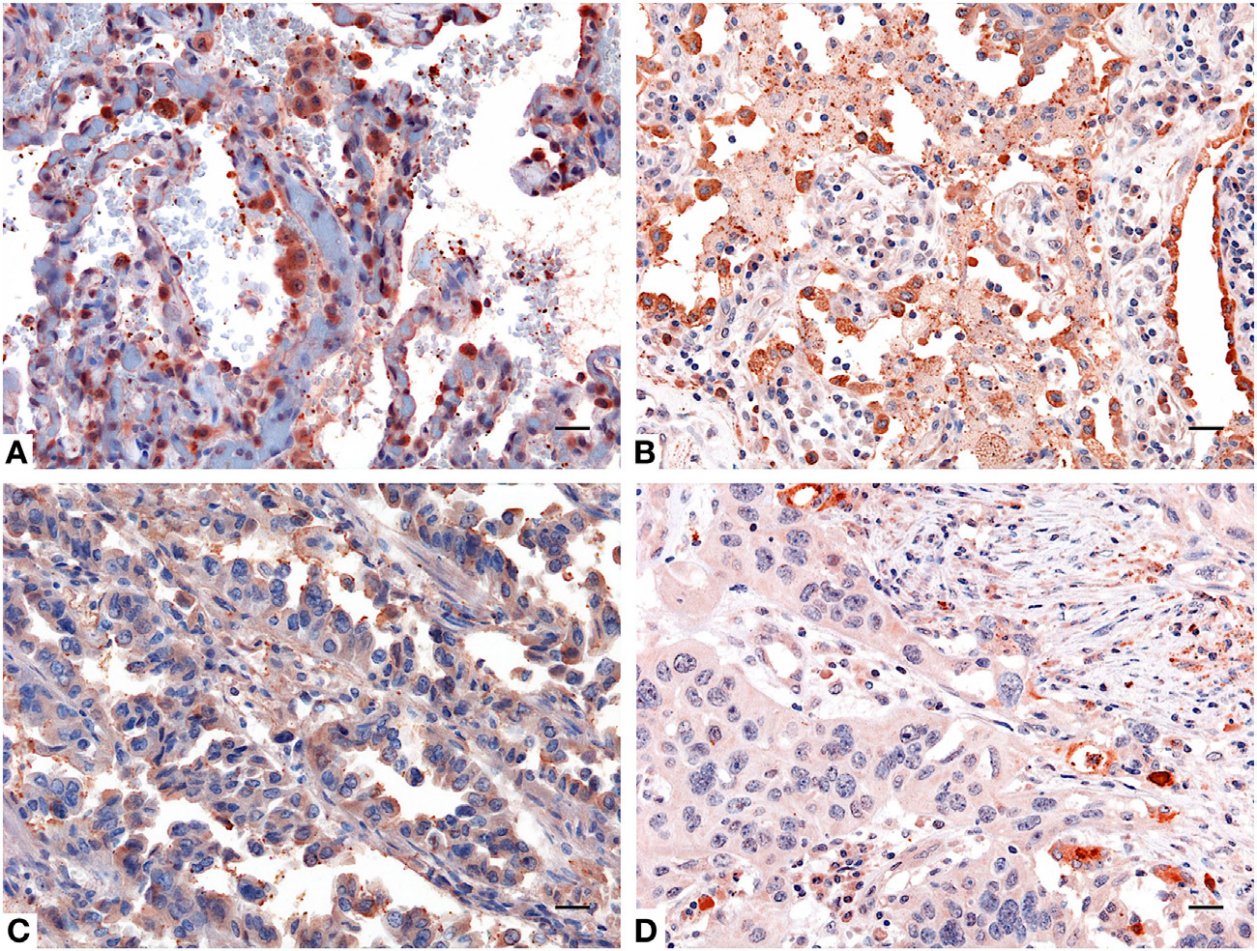
Immunohistochemistry analysis for surfactant protein D (SP-D) in lung. Compared to healthy lung **(A,B)** a decreased expression of SP-D in adenocarcinoma **(C)** and squamous cell carcinoma of the lung **(D)** was observed. Polymer detection system with AEC (red) chromogen was used; scale bars, 50 μm. Polymer detection system with AEC (red) chromogen was used; scale bars, 50 µm.

IHC staining for SP-D confirmed a differential expression in healthy and neoplastic pulmonary parenchyma. Moreover, in lung adenocarcinoma and squamous cell lung carcinoma tissues, we observed a lower expression of SP-D within its microenvironment compared to the healthy pulmonary parenchyma (Figure 2).

### Pathological Significance of SP-D mRNA Expression in Gastric, Breast, and Ovarian Cancers

**Figure 3:**
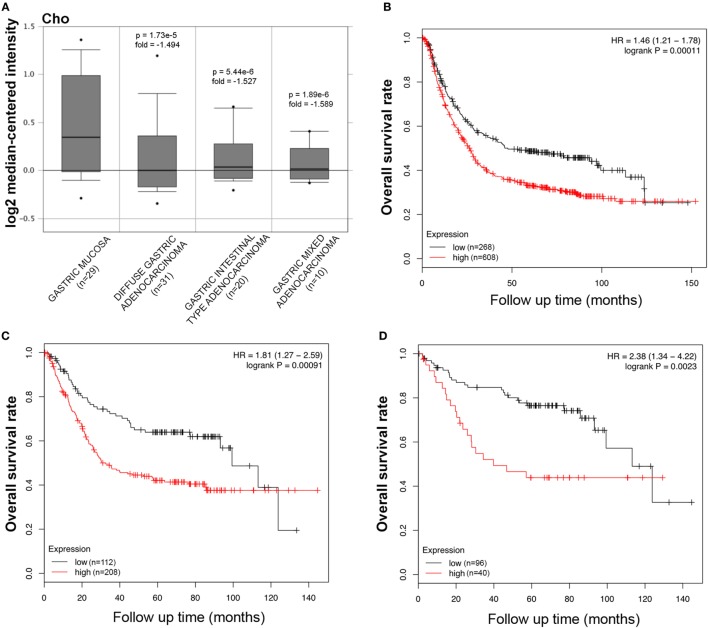
Pathological significance of SP-D expression in gastric cancer. Cho’s dataset has explored SP-D mRNA expression in gastric cancer. A lower *SFTPD* expression was detectable in gastric cancer than that in normal mucosa, even stratified into diffuse-, intestinal-, and mixed-type adenocarcinomas by Lauren’s classification [**(A)** *p* < 0.05]. According to the data from Kaplan–Meier plotter, SP-D mRNA expression was negatively related to an overall survival rate of the patients with gastric cancer [**(B)** *p* < 0.05]. If stratified by Lauren’s classification, SP-D mRNA expression was negatively related to an overall survival rate in the patients with intestinal-type adenocarcinoma [**(C)** *p* < 0.05], without distant metastasis and Her2-negative [**(D)** *p* < 0.05]. Abbreviations: HR, hazard ratio; SP-D, surfactant protein D.

The bioinformatics analysis on SP-D mRNA expression in gastric cancer *via* Cho’s and DErrico’s datasets showed its higher expression in healthy gastric mucosa compared to its malignant counterpart, stratified into intestinal, diffuse, and mixed-type adenocarcinoma by Lauren’s classification (Figure 3A, *p* < 0.05; Figure S2 in Supplementary Material, *p* < 0.05). According to the data from Kaplan–Meier plotter, SP-D mRNA expression was negatively related to an overall survival rate of the patients with gastric cancer (Figure 3B, *p* < 0.05). If stratified by Lauren’s classification, SP-D mRNA expression had a statistically significant association with intestinal-type adenocarcinoma, whereas no association with diffuse- and mixed-type adenocarcinomas was found (Figure 3C, *p* < 0.05). A higher expression of SP-D was negatively correlated with an overall survival rate in the patients without distant metastasis, HER2-negative and only intestinal-type adenocarcinoma (Figure 3D, *p* < 0.05).

**Figure 4:**
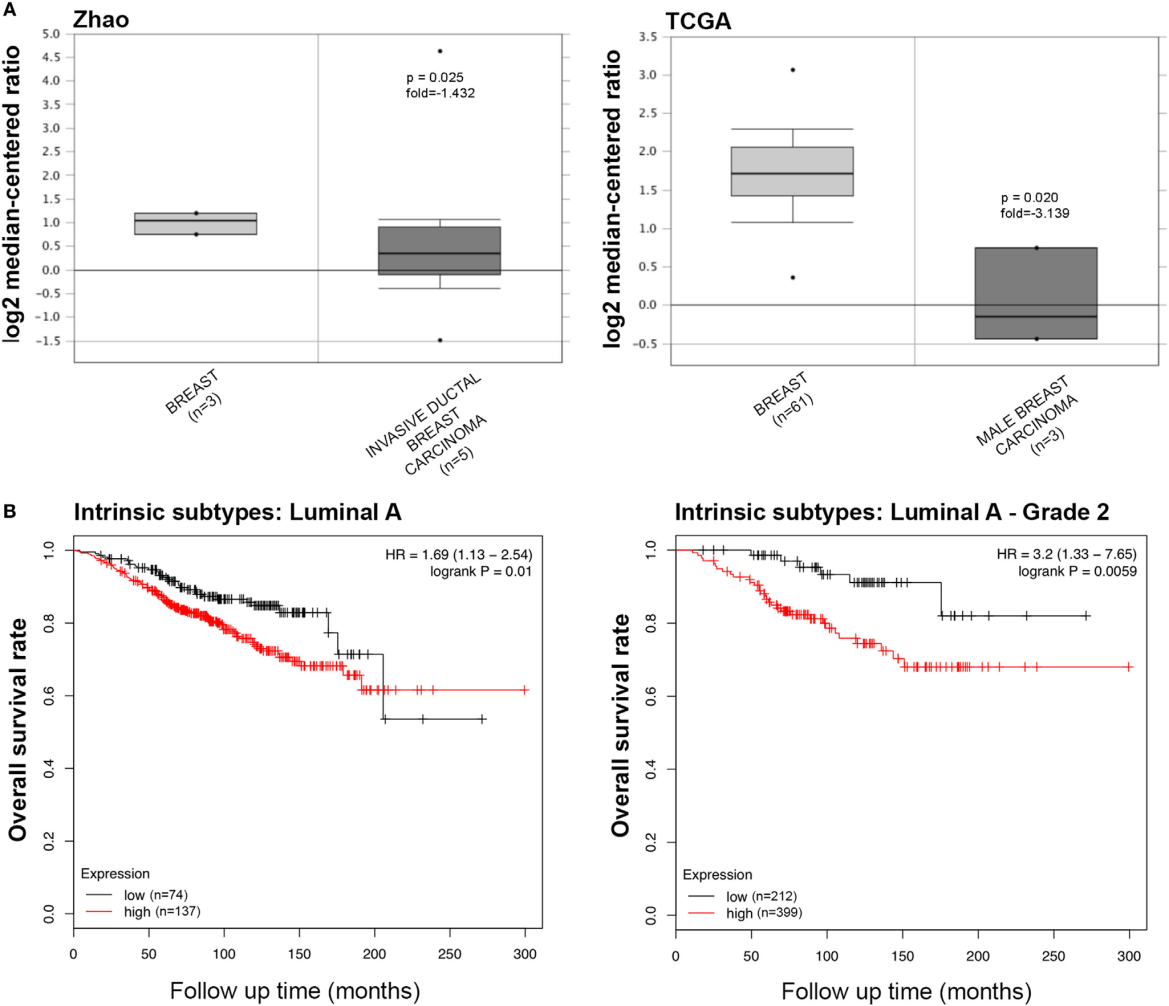
The clinicopathological significances of SP-D expression in breast cancer. Zhao’s and TCGA’s datasets have revealed a lower SP-D mRNA expression in invasive ductal breast carcinoma and male breast carcinoma than in normal breast tissues [**(A)** *p* < 0.05]. There was a negative association between SP-D mRNA expression and a favorable prognosis in the breast cancer patients with Luminal-A only with grade-1 and -2 cancers, for Kaplan–Meir plotter [**(B)** *p* < 0.05]. Abbreviations: HR, hazard ratio; SP-D, surfactant protein D.

The information regarding the SP-D mRNA expression in breast cancer was obtained from Zhao’s, TCGA’s, and Curtis’s datasets, which showed that *SFTPD* was expressed at a lower level in invasive ductal breast carcinoma, male breast carcinoma, and breast phyllodes tumor, compared to normal breast tissues (Figure 4A, *p* < 0.05; Figure S3A in Supplementary Material, *p* < 0.05). According to the data from Kaplan–Meir plotter, *SFTPD* expression was negatively linked to the high overall survival rate in breast cancer patients with Luminal-A grade-1 and grade-2 cancers (Figure 4B, *p* < 0.05; Figure S3B in Supplementary Material, *p* < 0.05). No correlation between SP-D mRNA expression and overall survival rate was observed in patients with the other characteristics (Luminal-B, HER2^+^, Basal, grade-3, mutated p53, wild-type p53).

**Figure 5:**
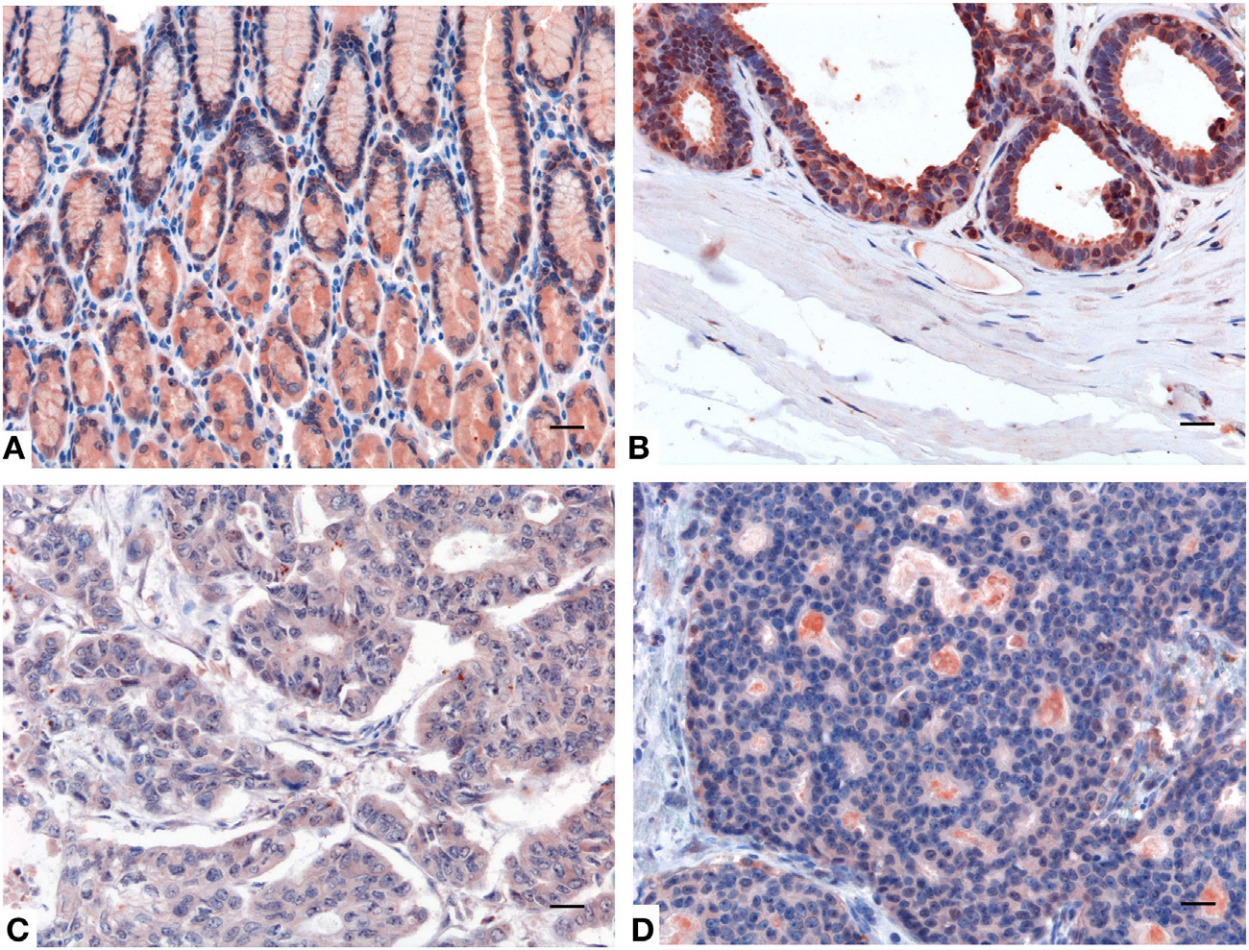
Representative immunohistochemical microphotographs of surfactant protein D (SP-D) expression in the healthy gastric mucosa **(A)**, and ductal mammary epithelium **(B)** and their malignant histotypes intestinal-type gastric adenocarcinoma **(C)** and invasive ductal breast carcinoma, Luminal-A **(D)**. A decreased expression of SP-D in the intestinal-type gastric adenocarcinoma and invasive ductal breast carcinoma, Luminal-A respect to their normal counterparts can be observed. Polymer detection system with AEC (red) chromogen; scale bars, 50 µm.

Using IHC, we observed a variable presence and distribution of SP-D in normal tissues with respect to their cancer counterpart. In fact, IHC performed on either healthy or neoplastic gastric mucosa highlighted a significantly reduced expression of SP-D in the intestinal-type adenocarcinoma compared to gastric control tissue (Figures 5A,C). Likewise, a higher expression of SP-D in the normal mammary parenchyma was detected compared to that observed within microenvironment within the invasive ductal breast carcinoma, Luminal-A (Figures 5B,D).

**Figure 6:**
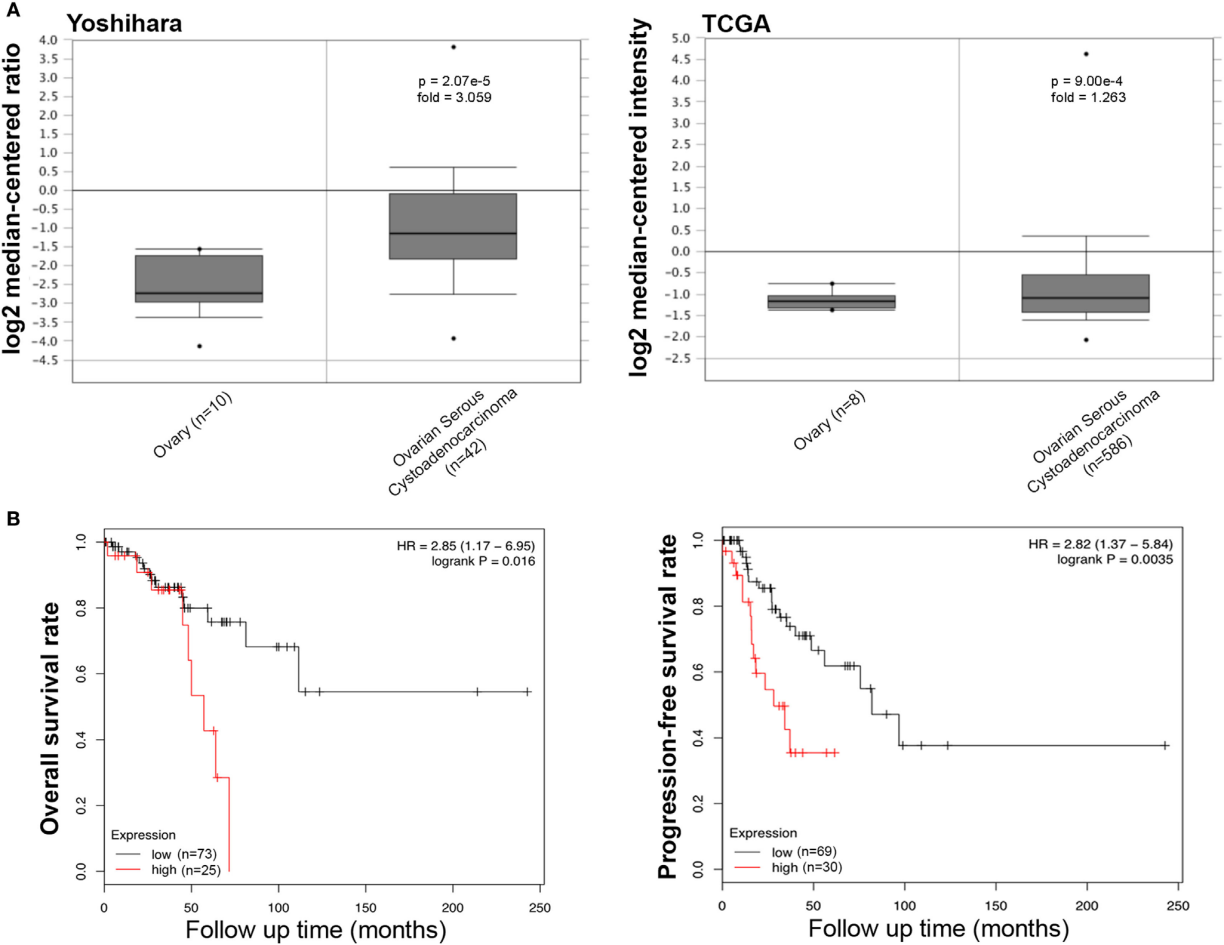
Pathological significance of SP-D expression in ovarian cancer. Yoshihara’s and TCGA’s datasets explored SP-D mRNA expression in ovarian cancer. A higher SP-D mRNA expression was detectable in serous cystadenocarcinoma than that in normal ovary [**(A)** *p* < 0.05]. According to the data from Kaplan–Meir plotter, SP-D mRNA expression showed a negative relationship both overall or progression-free survival rates of patients with serous cystadenocarcinoma, if stratified by stage-1 and -2 [**(B)** *p* < 0.05]. Abbreviations: HR, hazard ratio; SP-D, surfactant protein D.

We collected the results from Yoshihara’s and TCGA’s datasets and analyzed *SFTPD* expression in ovarian cancer. We observed a lower expression of *SFTPD* mRNA expression in normal ovary than in serous cystadenocarcinoma (Figure 6A, *p* < 0.05). The Kaplan–Meier plotter data, derived from stage-1 and -2 patients, showed a negative ratio between *SFTPD* expression and either overall or progression-free survival rates of patients with serous cystadenocarcinoma (Figure 6B, *p* < 0.05). However, no correlation was observed between *SFTPD* expression and these parameters (overall or progression-free survival rates) of patients with stage-3 and -4 ovarian cancer.

### SP-D Expression in the Microenvironment of Ovarian Cancer

**Figure 7:**
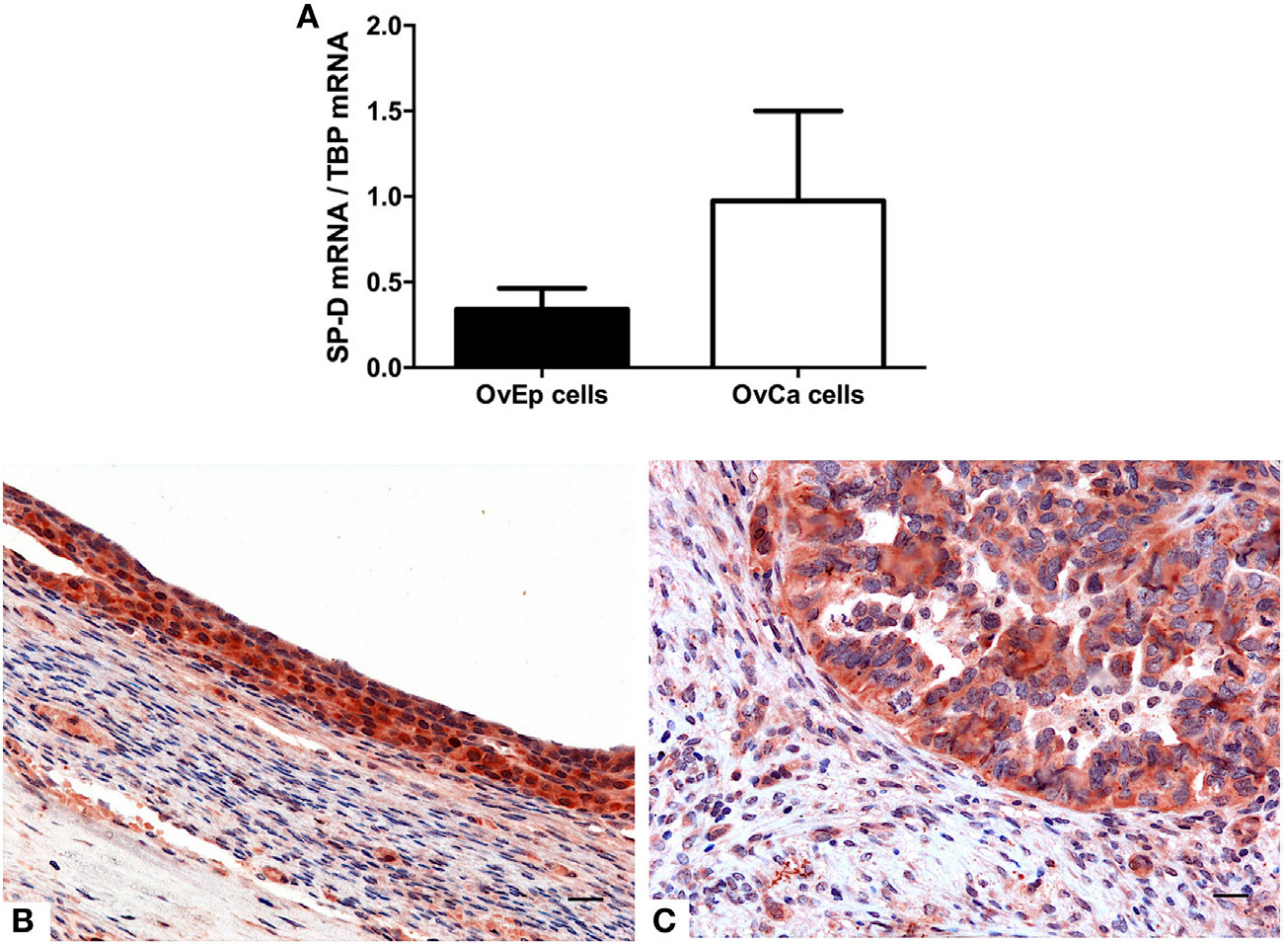
Quantitative real-time PCR analysis of surfactant protein D (SP-D) performed on the normal ovarian epithelium and the epithelial ovarian tumor **(A)**. Representative microphotographs showing an enrichment in SP-D positive cells in the serous cystadenocarcinoma **(B)** compared to the normal ovarian epithelium **(C)**. Polymer detection system with AEC (red) chromogen; scale bars, 50 µm.

The mRNA expression of SP-D was also evaluated by real-time PCR in primary cells isolated from four samples each of human ovarian serous cystadenocarcinoma and normal ovarian tissues. As shown in Figure 7A, the cells isolated from ovarian serous cystoadenocarcinoma tissues expressed more SP-D compared to the normal tissue, confirming the data obtained with the bioinformatics analysis. IHC analysis also revealed the presence and the distribution of SP-D in the normal ovary where it appeared to be localized in the ovarian epithelium lining and in the serous cystadenocarcinoma. In addition, we detected a differential expression in the normal as well as its malignant histotypes. Moreover, in the ovarian context, it showed an enrichment of SP-D expressing cells within the tumor microenvironment compared to the control tissue (Figures 7B,C).

## Discussion

The importance of SP-D in the regulation of the inflammation and homeostasis and in the protection against infection and allergens in the lung and at a range of extra-pulmonary mucosal sites is well documented (26, 27). However, there are recent evidences to implicate SP-D as an immune surveillance molecule against cancer (19, 20). In this study, we examined the potential prognostic value of this protein in lung, gastric, breast, and ovarian cancers. We focused our attention on these tumor types because we performed a bioinformatics analysis using the Kaplan–Meier plotter dataset, a manually curated database containing the information of 54,675 genes on 5,143 breast, 1,816 ovarian, 2,437 lung, and 1,065 gastric cancer samples. This is the most updated and reliable dataset available that offers the possibility of stratifying the analysis based on different tumor settings. The bioinformatics analysis highlighted a favorable prognostic effect of SP-D mRNA expression in the lung cancer, both in adenocarcinoma and squamous cell carcinoma; on the contrary, an unfavorable prognostic effect in gastric, ovarian, and breast cancer was revealed. In particular, SP-D mRNA expression showed a negative correlation with the intestinal-type gastric adenocarcinomas, grade-1 and grade-2 breast cancers and with stage-1 and -2 ovarian cancers. No significant correlation was showed within stage-3 and -4 in breast and ovarian cancers.

Sin et al. (28) have suggested that low SP-D levels may be correlated with the development of lung cancer. They observed a reduction of the concentration of SP-D in the bronchoalveolar lavage fluid of heavy smokers that was linked to bronchial dysplasia. More recently, Hasegawa et al. (19) noted the presence of SP-D in lung cancer, and demonstrated that SP-D was able to interfere, *via* its CRD region, with the interaction between EGF and EGF receptor (EGFR), a tyrosine kinase receptor of the ErbB family, causing downregulation of the EGF induced signaling (19). EGFR is commonly altered in epithelial tumors and its dysregulation leads to cell proliferation, angiogenesis, invasion, and metastasis (29). Furthermore, it has been recently demonstrated that SP-D is also able to interact with the mutant form of EGFR, inhibiting its ligand-independent dimerization (29). In addition, Kaur et al. have reported the ability of a recombinant truncated form of human SP-D to induce apoptosis *via* TNF-α/Fas-mediated pathway in human pancreatic adenocarcinoma using Panc-1 (p53^mt^), MiaPaCa-2 (p53^mt^), and Capan-2 (p53^wt^) cell lines. Treatment of these cell lines with a recombinant form of truncated human SP-D (made up of homotrimeric neck and C-type lectin domains) for 24 h caused growth arrest in G1 cell cycle phase and triggered transcriptional upregulation of pro-apoptotic factors such as TNF-α and NF-κB. Translocation of NF-κB from the cytoplasm into the nucleus of pancreatic cancer cell lines was observed following treatment with SP-D. SP-D treatment caused upregulation of pro-apoptotic marker Fas, which then triggered cleavage of caspase 8 and 3. This study raises the possibility of using recombinant SP-D as a therapeutic molecule against pancreatic cancer irrespective of their p53 phenotype (20).

The EGFR is commonly overexpressed in non-small cell lung cancer (in 89% squamous cell carcinoma; 41% adenocarcinomas) (30), and therefore, it is considered a potential target for cancer therapy (30). The presence of SP-D in these cancers could exert a protective effect *via* downregulation of the EGFR pathway. It has also been shown that serum level of SP-D reflects its levels in the lung and that higher amount of SP-D in the serum correlated with better overall survival in patients with EGFR mutant adenocarcinoma undergoing treatment with gefitinib, a tyrosine kinase inhibitor (29).

Our study appears to highlight a more favorable prognosis for adenocarcinoma with respect to squamous cell carcinoma. A possible explanation of this observation may be that adenocarcinoma originates from peripheral airways progenitor cells that are able to produce SP-D. Moreover, more SP-D production may be indicative of a more differentiated cancer.

*SFTPD*, together with a number of genes selectively expressed in the respiratory epithelial cells, is under the control of the thyroid transcription factor 1 (TTF-1) (31, 32). A recent meta-analysis showing that TTF-1 overexpression is related to a favorable prognosis for non-small cell lung carcinoma patients (33), appears to strengthen the results being reported here.

Although the overexpression of the *EGFR* gene has also been reported in a variety of other cancers including those of head and neck, ovary, cervix, bladder, esophagus, stomach, brain, breast, endometrium, and colon (24), the above-mentioned mechanisms cannot explain the opposite results obtained *via* the bioinformatics analysis of Kaplan–Mayer dataset for gastric, ovarian, and breast carcinomas, where SP-D showed an unfavorable prognostic effect. We think that the unfavorable prognostic effect of SP-D in other tumor settings can be due to its direct or indirect action on the immune population present in the tumor microenvironment (15). The following mechanisms can explain the role of SP-D in determining a tumor microenvironment favorable to tumor progression. For example, the protective effect of SP-D against breast cancer cells can be negated by the presence of hyaluronic acid, which is abundantly present in the microenvironment of a number of solid tumors (34) (Murugaiah, Bulla, and Kishore, unpublished data).

SP-D is able to reduce the expression of CD11c (15). CD11c is predominantly expressed on dendritic cells, but also on effector cells in the local tumor microenvironment, such as some macrophages, natural killer (NK), and activated T cells (25). It has been shown that low CD11c expression indicates unfavorable prognosis in patients with gastric cancer (35).

SP-D can promote production of TNF-α and IFN-γ (16, 18, 36). The anti-tumor effects of Th1 cells may reflect their known role in enhancing CD8^+^ T cell responses and activating macrophages, through the secretion of TNF-α and IFN-γ. IFN-γ can increase tumor cell class I MHC expression and sensitivity to lysis by NK cells and cytotoxic T lymphocytes (CTLs). Besides, antigen-presenting cells such as macrophages and dendritic cells can directly activate antigen-specific Th1 or CTLs, which can activate the anti-tumor immune response and are thus associated with favorable prognosis in a diverse range of cancers (37, 38).

It has been demonstrated that SP-D binds to lymphocytes and suppress T cell proliferation (14) *via* apoptosis induction in activated PBMCs. SP-D has been shown to enhance expression of CTLA-4, a negative regulator of T cell activation and proliferation (39). In addition, monocytes expressed CTLA-4, but only the lymphocytes treated with SP-D show a significant overexpression of CTLA-4 (15). There are strong experimental and clinical evidence to suggest that T cell responses to some tumors are inhibited by the involvement of CTLA-4, one of the best-defined inhibitory pathways in T cells (40, 41). In fact, tumor-infiltrating T cells often have a dysfunctional (exhausted) phenotype that is characterized by impaired effector functions and increased expression of CTLA-4 and other inhibitory molecules (40, 41). Blockade of the CTLA-4 pathways is now being widely used in the clinic to reverse the dysfunctional phenotype of tumor-specific T cells and enhance their ability to kill tumor cells (41). Thus, SP-D, by increasing the expression of CTLA-4, may contribute to the inhibition of the anti-tumor immune responses.

SP-D is able to inhibit the IL-12p40 production by macrophages *via* the SIRPα/ROCK/ERK signaling pathway (12). IL-12p40 is a component of IL-12p70 and IL-23, and its regulation is important for both innate and adaptive immunity. IL-12p40 is a marker of M1-like macrophages and data indicate that IL-12p40 may be contributing to inducing Th 1 polarization (42, 43). Macrophages derived from IL-12p40-deficient mice have a bias toward M2-like polarization (42). The production of IL-12p40 by macrophages and dendritic cells is associated with the ability to migrate to the lymph node and initiate T cell responses (44). We think that SP-D repressing the expression of IL-12p40 in macrophages may maintain the steady M2-like polarization and inhibit Th1 polarization.

SP-D has also been shown to interact with the leukocyte-associated Ig-like receptor-1 (LAIR-1) (45), known as CD305. This molecule is a transmembrane glycoprotein and is expressed on almost all immune cells as well as CD34^+^ hematopoietic progenitor cells. SP-D acts as a ligand for the inhibitory receptor LAIR-1, which inhibits the function of multiple types of immune cells (45), indicating that SP-D present in the tumor microenvironment may exert its immunomodulatory effect and inhibit the anti-tumor immune responses through LAIR-1 activation. Thus, the context of immune infiltration and composition of tumor microenvironment are likely to dictate the consequent effects of SP-D, and hence, tumor progression or resistance.

In summary, our *in silico* analysis, if confirmed with a retrospective study at the protein level, could highlight a possible role of SP-D as a novel marker for tumor prognosis in a range of cancers. The presence of SP-D could be associated with a favorable prognosis in lung cancer where it has been demonstrated to downregulate the EGF signaling, and unfavorable prognosis in non-pulmonary sites such as gastric, breast, and ovarian cancers.

## Ethics Statement

This study was carried out in accordance with the recommendations of governmental guidelines, and approved by the CEUR (Comitato Etico Unico Regionale, FVG, Italy) with written informed consent from all subjects, who gave written informed consent in accordance with the Declaration of Helsinki.

## Author Contributions

Conception and design: AM and RB. Development of methodology: AG, IF, CA, and FR. Acquisition of data: BB and CA. Analysis and interpretation of data (e.g., statistical analysis, biostatistics, and computational analysis): CT, FZ, AM, BB, and CA. Writing, review, and/or revision of the manuscript: RB, UK, CA, and GR. Study supervision: RB.

## Conflict of Interest Statement

The authors declare that the research was conducted in the absence of any commercial or financial relationships that could be construed as a potential conflict of interest. The reviewer AK declared a shared affiliation, though no other collaboration, with one of the authors UK to the handling Editor.

## Footnotes

^1^www.oncomine.org.

^2^www.kmplot.com.

## Supplementary Material

Figure S1 Surfactant protein D (SP-D) expression in lung cancer. Garber’s dataset has explored SP-D mRNA expression in the lung cancer. A lower SP-D mRNA expression was detectable in large cell carcinoma, adenocarcinoma, squamous cell carcinoma, and small cell carcinoma than in normal lung tissue (*p* < 0.05).

Figure S2 Surfactant protein D (SP-D) expression in gastric cancer. DErrico’s dataset has revealed a lower SP-D mRNA expression in intestinal-type adenocarcinoma than in normal mucosa (*p* < 0.05).

Figure S3 Pathological significance of SP-D expression in breast cancer. Curtis’s dataset has revealed a lower SP-D mRNA expression in phyllodes tumor than in normal breast tissue [**(A)** *p* < 0.05]. There was a negative association between SP-D mRNA expression and a favorable prognosis in the breast cancer patients with Luminal-A with grade-1, for Kaplan–Meir plotter [**(B)** *p* < 0.05]. Abbreviations: HR, hazard ratio; SP-D, surfactant protein D.
