# Supplementary figures and images for: Pathological Significance and Prognostic Value of Surfactant Protein D in Cancer
Source: Front Immunol. 2018 Aug 6;9:1748. doi: 10.3389/fimmu.2018.01748 (PMC6088209; doi:10.3389/fimmu.2018.01748)

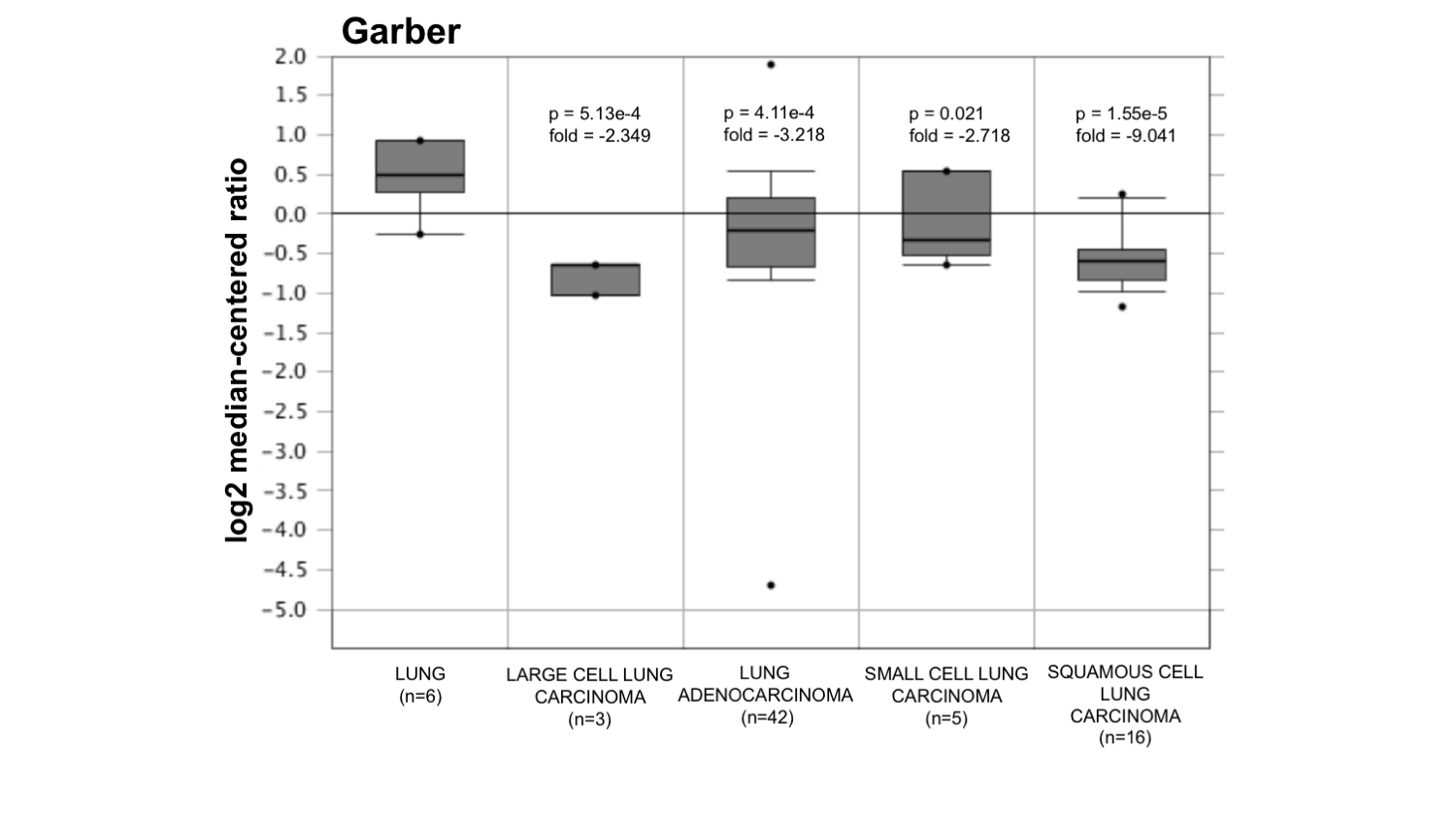

Supplement: Figure S1 — Surfactant protein D (SP-D) expression in lung cancer. Garber’s dataset has explored SP-D mRNA expression in the lung cancer. A lower SP-D mRNA expression was detectable in large cell carcinoma, adenocarcinoma, squamous cell carcinoma, and small cell carcinoma than in normal lung tissue (p < 0.05). [file image_1.tiff]

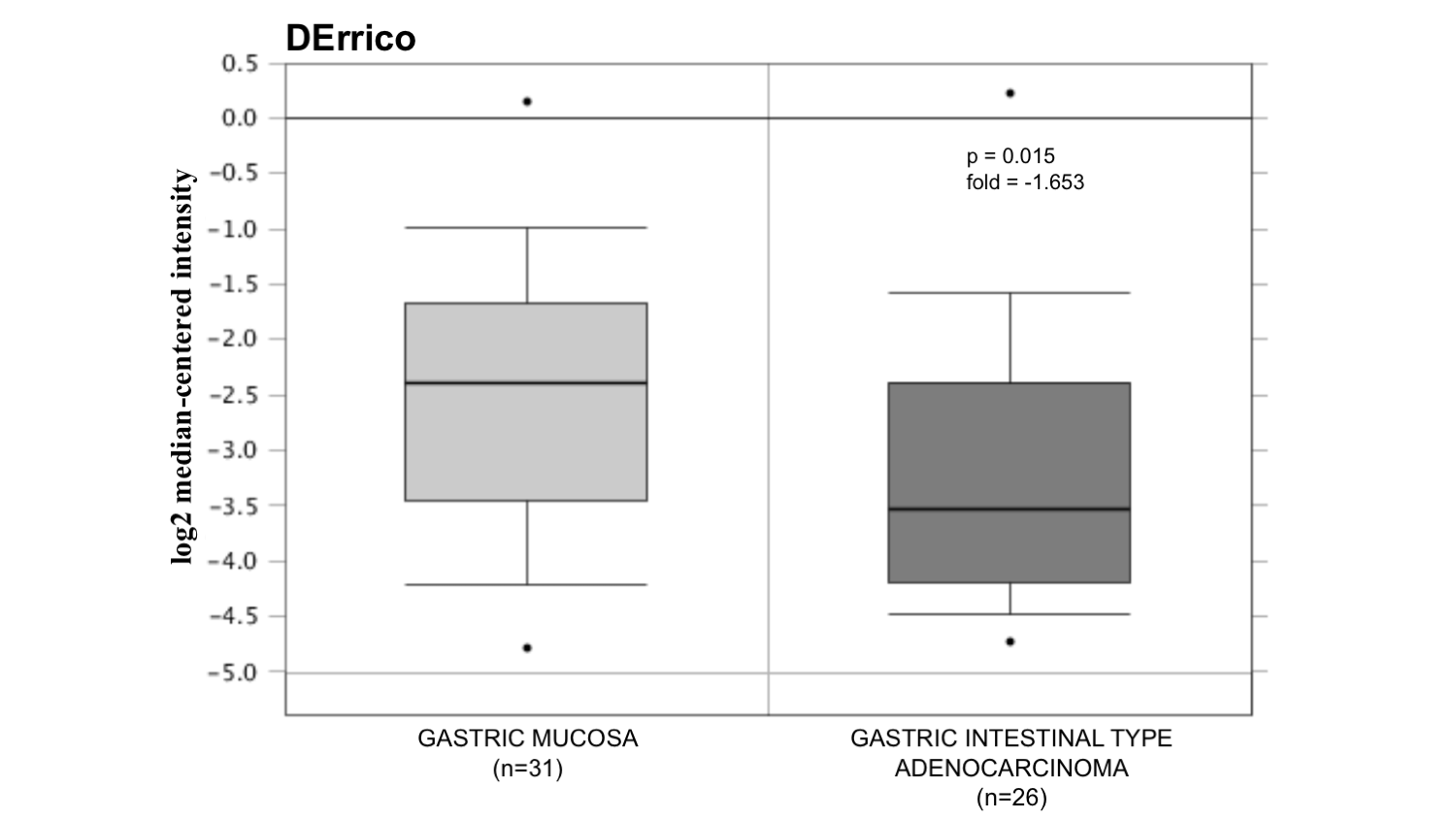

Supplement: Figure S2 — Surfactant protein D (SP-D) expression in gastric cancer. DErrico’s dataset has revealed a lower SP-D mRNA expression in intestinal-type adenocarcinoma than in normal mucosa (p < 0.05). [file image_2.tiff]

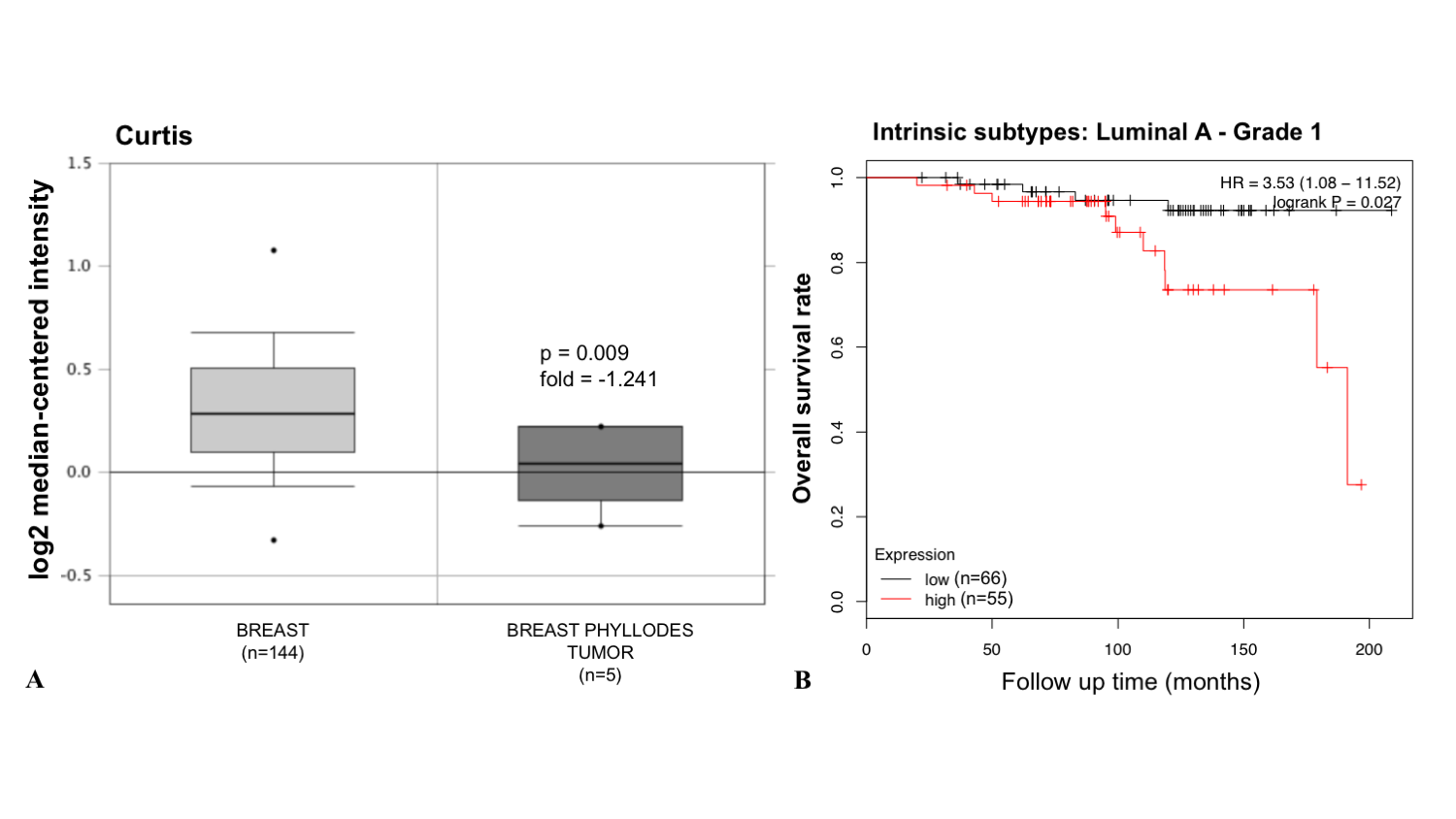

Supplement: Figure S3 — Pathological significance of SP-D expression in breast cancer. Curtis’s dataset has revealed a lower SP-D mRNA expression in phyllodes tumor than in normal breast tissue [(A) p < 0.05]. There was a negative association between SP-D mRNA expression and a favorable prognosis in the breast cancer patients with Luminal-A with grade-1, for Kaplan–Meir plotter [(B) p < 0.05]. Abbreviations: HR, hazard ratio; SP-D, surfactant protein D. [file image_3.tiff]
